# Direct Inference and Probabilistic Accounts of Induction

**DOI:** 10.1007/s10838-021-09584-0

**Published:** 2022-01-17

**Authors:** Jon Williamson

**Affiliations:** Department of Philosophy and Centre for Reasoning, University of Kent, Canterbury, UK

**Keywords:** Induction, Direct inference, Principle of the Narrowest Reference Class, Principal Principle, Bayesianism, Logical probability

## Abstract

Schurz (2019, ch. 4) argues that probabilistic accounts of induction fail. In particular, he criticises probabilistic accounts of induction that appeal to direct inference principles, including subjective Bayesian approaches (e.g., Howson 2000) and objective Bayesian approaches (see, e.g., Williamson 2017). In this paper, I argue that Schurz’ preferred direct inference principle, namely Reichenbach’s Principle of the Narrowest Reference Class, faces formidable problems in a standard probabilistic setting. Furthermore, the main alternative direct inference principle, Lewis’ Principal Principle, is also hard to reconcile with standard probabilism. So, I argue, standard probabilistic approaches cannot appeal to direct inference to explicate the logic of induction. However, I go on to defend a non-standard objective Bayesian account of induction: I argue that this approach can both accommodate direct inference and provide a viable account of the logic of induction. I then defend this account against Schurz’ criticisms.

## Introduction

There are two problems of induction. The more famous of the two, the problem of inductive justification, is the problem that there seems to be no justification of inductive inference which could convince sceptical detractors that we ought to draw even simple inductive inferences. This is the problem usually attributed to David Hume, and the problem over which most ink has been spilt. It has proven intractable: most philosophers would agree that there is indeed no such justification, although Schurz (2019) is more optimistic, arguing interestingly that metainduction offers a solution to Hume’s problem.

The second problem is perhaps the more pressing of the two. This is the problem of inductive logic—the problem that there seems to be no viable logic of inductive inference that can tell us which inductive inferences we ought to draw. This is the more pressing problem because inductive inferences are central to science and these inferences are often complex and contentious; a viable inductive logic could offer useful guidance. The problem of inductive justification, on the other hand, is largely academic: there are in fact no detractors who abstain from drawing all inductive inferences in practice.^1^ As Hume himself notes,

Whoever has taken the pains to refute the cavils of this *total* scepticism, has really disputed without an antagonist, and endeavour’d by arguments to establish a faculty, which nature has antecedently implanted in the mind, and render’d unavoidable. (Hume 1739, 183)

Again, most philosophers would agree that, following the demise of Carnap’s programme for inductive logic, a general inductive logic is unattainable. On the other hand, many would maintain that probabilistic approaches to induction are helpful in a range of cases. Schurz (2019, ch. 4) argues against probabilistic accounts of induction, however.

This paper focuses on the second, more pressing problem—the problem of inductive logic—though it also contains some brief remarks about the problem of inductive justification. The goal of the paper is to examine whether there is some viable probabilistic account of induction, and, if so, what that account is. I argue in Sect. 2 that *direct inference*, which requires that epistemic probabilities be directly calibrated to non-epistemic probabilities where possible, offers the most promising route to a probabilistic account of induction. There are two forms of direct inference. The Principle of the Narrowest Reference Class calibrates epistemic probabilities to generic frequencies, while the Principal Principle calibrates them to single-case chances. In Sect. 3 I consider a problem for the Principle of the Narrowest Reference Class which shows that in a standard probabilistic framework one cannot appeal to this direct inference principle to provide an adequate account of induction. In Sect. 4 I recount another problem, which shows that the standard probabilistic framework fails to properly accommodate the Principal Principle. Thus the standard probabilistic framework cannot employ direct inference to account for induction after all. However, I argue that a non-standard version of objective Bayesianism can successfully accommodate direct inference (Sect. 5). I counter Schurz’ criticisms of objective Bayesianism in Sect. 6 and conclude that it does indeed provide a viable probabilistic account of the logic of induction (Sect. 7).^2^

## The Promise of Direct Inference

The standard probabilistic response to the inductive logic problem proceeds from one of two general frameworks: that of Bayesianism or that of logical probability.

In the Bayesian framework, probabilities are epistemic—they are an agent’s rational degrees of belief. If ${\mathcal {L}}$ is the agent’s language, in which she can express propositions *A* and *E*, we denote by $B_E(A)$ her degree of belief in *A*, supposing only *E*. The standard Bayesian approach maintains that all conditional beliefs are conditional probabilities:

- *CBCP.* There is a probability function $P_{\emptyset }$ such that $B_E(A) = P_{\emptyset }(A|E)$ for all *A* and *E*.

This motivates the principle of Bayesian Conditionalisation, which governs how degrees of belief should track evidence:

- *Conditionalisation.* On evidence *E*, believe *A* to degree $B_E(A)= P_{\emptyset }(A|E)$.

Here the *prior* probability function $P_{\emptyset }$ is either a function that is appropriate in the total absence of evidence (a *blank-slate* function) or a probability function that was appropriate at some initial time, evidence prior to which is not made explicit (an *initial* function).

According to logical probability, on the other hand, probability is fundamentally a relation between propositions. If ${\mathcal {L}}$ is a language that is appropriate to the problem domain, in which *A* and *E* are expressible propositions, we denote by $C_E(A)$ the degree to which *E* confirms *A*. The logical theory of probability maintains that degrees of conditional confirmation are conditional probabilities:

- *CCCP.* There is a probability function $P_{\emptyset }$ such that $C_E(A) = P_{\emptyset }(A|E)$ for all *A* and *E*.

Here the prior $P_{\emptyset }$ is a blank-slate function. Most proponents of logical probability, such as Keynes (1921) and Carnap (1950), hold that logical probabilities underwrite rational degrees of belief: $B_E(A)$ is rational just if $B_E(A) = C_E(A)$ for some appropriate confirmation function $C_E$.^3^

Although these two general frameworks differ as to the fundamental nature of probability, they broadly agree about three things: firstly, the need for an appropriate prior probability function $P_{\emptyset }$; second, that conditional probabilities play an absolutely central role; and third, that these conditional probabilities $P_{\emptyset }(A|E)$ underwrite rational degrees of belief.

There are three main implementations of these two general approaches.

*Strictly subjective Bayesianism* (de Finetti 1937; Howson 2000) maintains that any probability function $P_{\emptyset }$ constitutes a rationally permissible prior: it is up to the individual as to which such function her beliefs conform to via CBCP. Unfortunately, this approach does not seem to help much with the inductive logic problem. Consider a simple example. Suppose we randomly sample 21-year-olds and infer that 17% of 21-year-olds develop a cough in the next 12 months (proposition *E*). How confident should we be that Cheesewright, who is 21, will get a cough in the next year (*A*)? According to strictly subjective Bayesianism, any degree of belief in *A* is rationally permissible, since any prior is permissible. Even where we have the full details of the sample—i.e., if we know which 21-year-olds were sampled and which developed a cough—rather than just the inferred frequency of coughs, it is still the case that any degree of belief would be rationally permissible. Since the account claims that any degree of belief in *A* is rationally permissible, it does not provide a logic of induction, because it admits both beliefs that can be considered inductive (e.g., $B_E(A) \approx 0.17$) and beliefs that are non-inductive (e.g., the belief $B_E(A)=P_{\emptyset }(A)$ which is not influenced by the sample at all). At best then, strictly subjective Bayesianism merely provides a conditional logic of induction: only if one conforms to a prior that permits induction does it provide an account of learning from experience (Howson 2000). For similar reasons, strictly subjective Bayesianism cannot be said to solve the problem of inductive justification, because it deems both inductive and non-inductive priors to be rationally permissible and provides no grounds for preferring the former over the latter. At best it merely provides a conditional justification of induction: only if one adopts an inductive prior should one learn inductively from experience.

The second specific approach is an implementation of logical probability: *Carnap’s programme*. Carnap (1952) sought to objectively constrain the choice of the blank-slate function $P_{\emptyset }$. Now, by far the most natural choice of $P_{\emptyset }$ is the equivocator function $P_=$, which gives each state description the same probability (Williamson 2017, ch. 4).^4^ As Carnap realised, however, the equivocator function is non-inductive: CCCP ensures that there is no learning from experience if $P_{\emptyset }=P_=$, because $C_E(A)=P_=(A|E) =P_=(A)=0.5$ for an atomic sentence *A* and logically independent evidence *E*. So Carnap instead opted for a continuum of permissible blank-slate functions, $P_{\emptyset }= c_\lambda$, parameterised by a constant $\lambda \in [0,\infty )$, each of which does allow learning from experience.

Carnap’s programme offers more guidance than strictly subjective Bayesianism, but still does not help much with the problem of inductive logic. Although Carnap dismissed the non-inductive equivocator function, his account is nevertheless very permissive: the degree to which the sample of 21-year-olds confirms the proposition that Cheesewright will get a cough can be anywhere between 0.17 and 0.5. Carnap provides no clear guidance as to which value to opt for. Nor does Carnap’s programme help to address the problem of inductive justification. This is because the exchangeability condition—a key condition to which Carnap appeals to help narrow down the blank-slate functions to a continuum—is not universally appropriate. As Gillies (2000, 77–83) explains, this condition is usually only appropriate in the context of a sequence of outcomes that are believed to be objectively probabilistically independent. Thus Carnap’s programme cannot provide a general justification of induction.^5^

The third specific approach, *empirically-based Bayesianism*, is a version of Bayesianism that employs a direct inference principle in addition to the usual axioms of probability. A direct inference principle requires that degrees of belief be directly calibrated to non-epistemic probabilities insofar as one has evidence of them. There are two variants of this approach. One variant takes non-epistemic probabilities to be generic frequencies or propensities, and maintains that one should calibrate a degree of belief to a generic probability in a suitable reference class of individuals. The other variant takes non-epistemic probabilities to be single-case chances, and maintains that one should calibrate a degree of belief to such a chance. Prima facie, this approach offers a more promising way of tackling the problem of inductive logic. According to this approach, one should believe that the 21-year-old Cheesewright will get a cough to degree 0.17 or thereabouts, given a sample that warrants the inference that approximately 17% of 21-year-olds get a cough (*E*). This, at least, is concrete inductivist advice, although how concrete it is depends on what can be said about exactly how close to 0.17 one’s degree of belief should be.

This third approach also takes a more promising line with regard to the problem of inductive justification: some suitable justification of the direct inference principle might convince any detractor who accepts the existence of non-epistemic probabilities of the merits of inductive inference. Admittedly, someone sceptical about whether a sample should guide our inferences about an unsampled individual might also be sceptical about the claim that there are non-epistemic probabilities. Nevertheless, depending on how the details are fleshed out, this line of argument promises to make some modest headway with the problem of inductive justification.

So, while there are gaps in the account that need to be filled, some version of empirically-based Bayesianism that appeals to a direct inference principle seems to offer the most promise with regard to the two problems of induction. But which version of empirically-based Bayesianism? Not any standard version: as I argue in Sect. 3, 4, both standard Bayesianism and logical probability struggle to accommodate either of the two kinds of direct inference principle in a way that can secure induction. However, we shall see that there is one non-standard version of Bayesianism that does not succumb to these problems and that can fill the gaps identified above, offering an account of inductive logic and making some progress with the problem of inductive justification (Sect. 5, 6).

## The Principle of the Narrowest Reference Class

The idea of direct inference dates back at least to Leibniz (Cussens 2018). Leibniz took probability to be an epistemic concept at root: probabilities are degrees of certainty (Hacking 1975, 89). And Leibniz says,

One may still estimate likelihoods a posteriori, by experience; to which one must have recourse in default of a priori reasons. For example, it is equally likely that a [particular] child should be born a boy or a girl, because the number of boys and girls is very nearly equal all over the world. (Leibniz 1714, 570)

We normally perform direct inference without thinking about it—indeed, research conducted by Bastos and Taylor (2020a, b) suggests that even parrots can perform a fairly sophisticated form of direct inference. However, the question arises as to whether there is some explicit direct inference principle that can guide induction in more complex situations. Reichenbach put forward one such principle: a principle for using generic frequencies to guide single-case inferences:

We then proceed by considering the narrowest class for which reliable statistics can be compiled. If we are confronted by two overlapping classes, we shall choose their common class. Thus, if a man is 21 years old and has tuberculosis, we shall regard the class of persons of 21 who have tuberculosis. (Reichenbach 1935, 374)

This policy has become known as the Principle of the Narrowest Reference Class. The principle is intuitively plausible and widely endorsed—Schurz (2019, 58) is one recent advocate, for example. A simple version of the principle can be expressed as follows:^6^

- *PNRC*. $B_E(\alpha (c)) = x$ if *E* determines that the frequency $P^*_{\hat{\rho }}(\alpha )=x$ and determines that $\hat{\rho }$ is the unique narrowest reference class containing *c* for which $P^*_{\hat{\rho }}(\alpha )$ is available, and contains no information more pertinent to $\alpha (c)$.

Here $\alpha$ and $\rho$ denote properties and *c* denotes an individual. A property such as $\rho$ determines a reference class $\hat{\rho }$, namely the set of individuals that instantiate the property $\rho$. $P^*_{\hat{\rho }}(\alpha )$ is the frequency of $\alpha$ in the reference class $\hat{\rho }$. Clearly, something further needs to be said about when other information is more pertinent to $\alpha (c)$ than the frequency information, and we shall return to this point below.

Although PNRC is intuitively plausible, I will argue that it fails to yield an adequate account of induction, when integrated into a standard probabilistic framework such as the standard Bayesian framework or that of logical probability.

Let us consider some consequences of PNRC in the standard Bayesian framework, which takes conditional degrees of belief to be conditional probabilities (CBCP).^7^ Let proposition *A* abbreviate $\alpha (c)$; for example, *A* might be the proposition that Cheesewright gets a cough in the next year. Let *R* abbreviate $\rho (c)$, e.g., Cheesewright is 21. Let *S* stand for $\sigma (c)$, e.g., Cheesewright has tuberculosis. Let *X* be $P^*_{\hat{\rho }}(\alpha )=x$, e.g., the statement that the frequency of coughs in the reference class of 21-year-olds is 0.17. Let *Y* be $P^*_{\widehat{\rho \sigma }}(\alpha )=y$, where $y > x$, e.g., the statement that the frequency of coughs in the reference class of 21-year-olds with tuberculosis is 0.97.

Then PNRC apparently leads to the following inferences about Cheesewright:

1.: $$P_{\emptyset }(A|XR) = 0.17$$
2.: $$P_{\emptyset }(A|YRS) = 0.97$$
3.: $$P_{\emptyset }(A|XYR) = 0.17$$
4.: $$P_{\emptyset }(A|XYRS) = 0.97$$
5.: $$P_{\emptyset }(A|XYR\bar{S}) = 0.17$$.

Note that, for these conditional probabilities to be well defined, the probabilities of the propositions conditioned on must be non-zero. Thus these uses of PNRC presuppose that that $P_{\emptyset } (XYRS)>0$ and $P_{\emptyset }(XYR\bar{S}) >0$.

These implications of PNRC are all very natural. Indeed, it is hard to see what specific values other than these an inductivist would advocate in these five alternative scenarios. However, it turns out that these consequences of PNRC are inconsistent.^8^

To see this, observe that by the theorem of total probability,

i $$\begin{aligned} P_{\emptyset }(A|XYR) = P_{\emptyset }(A|XYRS) P_{\emptyset }(S|XYR) + P_{\emptyset }(A|XYR\bar{S}) P_{\emptyset }(\bar{S}|XYR) \end{aligned}$$

i.e.,

$$\begin{aligned} 0.17 = 0.97 s + 0.17 (1-s), \end{aligned}$$

where $s{\mathop {=}\limits ^{\mathrm{df}}} P_{\emptyset }(S|XYR)$. But this can only hold if $s=0$, which contradicts the presupposition that $P_{\emptyset }(XYRS)>0$.

This poses a problem for the Bayesian: it seems that Bayesianism cannot accommodate even a very simple version of the Principle of the Narrowest Reference Class, namely PNRC. This is essentially because standard Bayesianism assumes CBCP, which turns consequences of PNRC into constraints on a single probability function, namely the prior $P_{\emptyset }$, and these constraints soon become unsatisfiable. Note that exactly the same problem would arise in the general framework of logical probability, because it assumes the analogous principle CCCP. This poses a problem for standard probabilistic accounts of induction that appeal to PNRC or one of its generalisations.

Let us examine the options for such accounts. Is there some way of avoiding the problem?

As Equation i shows, the inconsistency is generated by consequences 3, 4 and 5 of PNRC:

3.: $$P_{\emptyset }(A|XYR) = 0.17$$
4.: $$P_{\emptyset }(A|XYRS) = 0.97$$
5.: $$P_{\emptyset }(A|XYR\bar{S}) = 0.17$$.

The inconsistency would be avoided if one were able to deny that PNRC yields all three of these three constraints on the prior. This would be possible if, in at least one of these three cases, what is conditioned upon contains information that can be deemed more pertinent to *A* than the frequency that is being used to inform the degree of belief. If so, PNRC would not apply in that case and no contradiction would be derivable: the probabilistic account would be able to accommodate PNRC, after all.

However, we shall see that denying any of these three conditions would be problematic because PNRC would then be very easily defeated. I will go on to argue that this problem shows that one cannot, after all, appeal to PNRC to provide an adequate probabilistic account of induction.

First consider consequence 3. One can reject this identity if one can deem *Y* to be more pertinent to *A* than *XR*, i.e., if one can deem the frequency in a narrower reference class to be more pertinent to *A* than that in a wider reference class, even where there is evidence only that the individual is a member of the wider reference class—not the narrower reference class. There are several difficulties with this strategy. Firstly, it conflicts with the idea behind PNRC, which is to calibrate a degree of belief to the frequency in the narrowest reference class that is known to apply to the particular individual. Second, it severely limits the applicability of PNRC, because we almost always do have superfluous frequency data that are of questionable relevance to an individual of interest. If these data defeat PNRC, then direct inference would appear to be rarely warranted.

Worse still, a mischief maker would be able to undermine *any* particular application of PNRC by reliably informing the agent seeking to apply PNRC of some statistic that is of dubious relevance. You might intend to use PNRC to calibrate your degree of belief that Cheesewright gets a cough to the frequency of coughs within some reference class that includes Cheesewright. The mischief maker then tells you that 35% of 21-year-olds who have COVID-19 develop a cough, where it is unknown to you whether Cheesewright has COVID-19. That would be enough to undermine your use of PNRC, if the above strategy is pursued. Not only would the proponent of probabilistic induction fail to convince a detractor of the merits of induction, but a mischievous detractor would be able to undermine the proponent’s own use of induction.

Let us turn next to consequence 4. To reject the identity $P_{\emptyset }(A|XYRS) = 0.97$, one would have to maintain that statistics in wider reference classes are more pertinent to an individual than those in narrower reference classes: *X* defeats *YRS*. This strategy would even more blatantly conflict with the aim of PNRC. As with the previous strategy, this would also render PNRC impotent, because it would be enough for the mischief maker to report the frequency of coughs in people of all ages in order to undermine any use of PNRC on some narrower reference class.

Suppose, then, that we grant consequences 3 and 4. Now consider consequence 5. Rejecting the identity $P_{\emptyset }(A|XYR\bar{S}) = 0.17$ would require claiming that a narrower reference class frequency defeats a wider reference class frequency in cases where the individual is not a member of the narrower reference class. Now, while consequence 5 is prima facie plausible, it turns out that one can indeed provide some grounds for rejecting it, along the following lines. Suppose frequencies are conditional probabilities, so that $P^*_{\hat{\rho }}(\alpha ) = P^*(\alpha |\rho )$ etc.^9^ Then,

ii $$\begin{aligned} P^*(\alpha |\rho ) = P^*(\alpha |\rho \sigma ) P^*(\sigma |\rho ) + P^*(\alpha |\rho \bar{\sigma }) P^*(\bar{\sigma }|\rho ) \end{aligned}$$

which we can write as:

$$\begin{aligned} x = y t + z (1-t), \end{aligned}$$

where $$x{\mathop {=}\limits ^{\mathrm{df}}}P^*(\alpha |\rho ), y{\mathop {=}\limits ^{\mathrm{df}}} P^*(\alpha |\rho \sigma ), z{\mathop {=}\limits ^{\mathrm{df}}} P^*(\alpha |\rho \bar{\sigma })$$ and $$t{\mathop {=}\limits ^{\mathrm{df}}} P^*(\sigma |\rho )$$.

In the Cheesewright example, $\alpha$ is the attribute getting a cough in the next year, $\rho$ is the reference class of 21-year-olds, $\sigma$ is the reference class of those with tuberculosis, $x=0.17$, $y=0.97$, and we have

$$\begin{aligned} 0.17 = 0.97 t + z (1-t). \end{aligned}$$

Assuming that $t>0$, this equation can only hold if $z < 0.17$. It follows that $P_{\emptyset }(A|XYR\bar{S})$ should be less than 0.17, contra consequence 5. One can see this as follows. Let us refer to the claim that $P^*(\alpha |\rho \bar{\sigma }) <0.17$ as proposition *Z*. As we have just seen, *Z* follows from *XY*. For each $z\in [0,1]$, let $Z_z$ be the claim that $P^*(\alpha |\rho \bar{\sigma })=z$. Then,

$$\begin{aligned} P_{\emptyset }(A|XYR\bar{S})= & {} \quad P_{\emptyset }(A|XYZR\bar{S})\\= & {} \int _{0}^{1} P_{\emptyset }(A|XYZR\bar{S} Z_z) P_{\emptyset }(Z_z|XYZR\bar{S}) \,\, {dz}\\= & {} \int _{0}^{0.17} z P_{\emptyset }(Z_z|XYZR\bar{S}) \,\, {dz}\\< & {} 0.17 \end{aligned}$$

since $P_{\emptyset }(Z_{0.17}|XYZR\bar{S})=0$. Note that the third equality holds by an application of PNRC: $P_{\emptyset }(A|XYZR\bar{S} Z_z) = P^*(\alpha |\rho \bar{\sigma }) = z$ for $z\in [0,0.17)$, or 0 for $z\in [0.17,1]$. So, if we assume that frequencies are conditional probabilities and that $P^*(\sigma |\rho )>0$, we reach the conclusion that $P_{\emptyset }(A|XYR\bar{S})<0.17$. In which case, consequence 5 cannot hold: $Y\bar{S}$ provides pertinent evidence that defeats the attempt to apply PNRC. This provides some reason to think that $Y\bar{S}$ is a defeater.

However, admitting $Y\bar{S}$ as a defeater opens the door to our mischief maker to undermine *any* application of PNRC, as we shall now see.

Suppose we have evidence *XR* and wish to apply PNRC to set $P_{\emptyset }(A|XR)=x$, where $0<x<1$. Then our mischief maker reliably informs us that there are some features $\sigma$ that are positively relevant to $\alpha$ but which do not all apply to individual *c*. (In real scenarios, there will always be some such features. We do not need to know precisely what the features are, nor precisely how relevant they are.) That $\sigma$ makes a positive difference to $\alpha$ is captured by the following proposition *Y*:

$$\begin{aligned} (P^*_{\widehat{\rho \sigma }}(\alpha ) > x) \wedge (P^*_{\widehat{\rho \bar{\sigma }}}(\alpha ) < x). \end{aligned}$$

That $\sigma$ does not apply to *c* is captured by the proposition $\bar{\sigma }(c)$, abbreviated by $\bar{S}$. Given the evidence $Y\bar{S}$ provided by the mischief-maker, one needs to consider $P_{\emptyset }(A|XYR\bar{S})$ instead of $P_{\emptyset }(A|XR)$. And it turns out that if we reject consequence 5 then $Y\bar{S}$ is a defeater here: it prevents us from using PNRC to set $P_{\emptyset }(A|XYR\bar{S})=x$. This is because the theorem of total probability forces $P_{\emptyset }(A|XYR\bar{S}) < x$, even where the difference $\sigma$ makes is unknown. To see this, let $Z_z$ be the proposition $P^*_{\widehat{\rho \bar{\sigma }}}(\alpha )=z$. Then,

$$\begin{aligned} P_{\emptyset }(A|XYR\bar{S})= & {} \int _z P_{\emptyset }(A|XYR\bar{S} Z_z) P_{\emptyset }(Z_z|XYR\bar{S}) \,\, {dz} \\= & {} \int _{0}^{x} z P_{\emptyset }(Z_z|XYR\bar{S}) \,\, {dz} \\< & {} x \end{aligned}$$

since $$P_{\emptyset }(Z_x|XYR\bar{S})=0$$.

Let us return to our specific example. Given *XR*, which says that 17% of 21-year-olds get a cough in the next year and Cheesewright is a 21-year-old, we might want to use PNRC to directly infer a degree of belief 0.17 that Cheesewright gets a cough. But then we are reliably informed that there is some set of factors that do not apply to Cheesewright but make a positive difference to the proposition that he gets a cough. Although this information is hardly surprising, if one rejects consequence 5 then this information alone is sufficient to defeat our use of PNRC, even if we can neither specify the factors nor the difference they make.^10^ This makes PNRC all too easy to undermine. The only way to render PNRC more robustly applicable is to insist on consequence 5—a move that blocks these mischievous defeaters.

We thus have a dilemma. If we affirm consequence 5, which has the merit of being intuitively plausible, we block the mischief maker. However, this comes at the expense of inconsistency: consequences 3-5 cannot all hold together. On the other hand, if we reject consequence 5, in line with the argument from Equation ii, then we enable the mischief maker to undermine even the simplest use of PNRC. This last point also goes for consequences 3 and 4: if we deny any of 3-5, we permit the mischievous inductive detractor to undermine our own inductive inferences.

The Bayesian would normally be inclined to take the second horn of the dilemma here, suggesting that it is no matter that PNRC is so easily undermined, as long as we take all potential underminers into account by employing the theorem of total probability. Such a response might proceed as follows. Suppose for simplicity that there are only finitely many properties that define reference classes, namely $\rho ,\sigma _{1},\ldots ,\sigma _{k}$. Let $S_{1},\ldots , S_{2^k}$ be propositions predicating all the various combinations of $\sigma _{1},\ldots ,\sigma _{k}$ applied to *c*, e.g., $\sigma _{1}(c)\bar{\sigma }_2(c)\cdots \bar{\sigma }_{k-1}(c)\bar{\sigma }_k(c)$. For each such $S_{i}$, let $Z_z^i$ be the claim that the frequency of $\alpha$ is *z* in the reference class picked out by $\rho$ together with the combination of properties appearing in $S_{i}$, e.g., $P^*_{\widehat{\rho \sigma _{1}\bar{\sigma }_2\cdots \bar{\sigma }_{k-1}\bar{\sigma }_k}}(\alpha )=z$. Note that such reference classes are narrowest reference classes. Then the Bayesian would require just that:

$$\begin{aligned} P_{\emptyset }(A|XR)= & {} \sum _{i=1}^{2^k} \int _z P_{\emptyset }(A|X R Z_z^i S_i) P_{\emptyset }(Z_z^i S_{i}|XR) \,\, {dz}\\= & {} \sum _{i=1}^{2^k} \int _z z P_{\emptyset }(Z_z^i S_{i}|XR) \,\, {dz} \end{aligned}$$

where the second identity follows by PNRC.

While this approach is perfectly in accord with the standard Bayesian framework, it is not an adequate response in this context because it undermines the appeal to PNRC to provide an adequate account of induction. The problem is that this approach does not commit to any precise value for $P_{\emptyset }(A|XR)$. Indeed, any value in the unit interval is deemed rationally permissible for $P_{\emptyset }(A|XR)$, as long as the values $P_{\emptyset }(Z_z^i S_{i}|XR)$ are set accordingly. So, when it comes to providing an account of induction, this approach suffers from exactly the same problems that beset strictly subjective Bayesianism: inductive inference and non-inductive inference are placed on an equal footing. What we wanted from an appeal to PNRC was to force a value of 0.17 or thereabouts for $P_{\emptyset }(A|XR)$, to show how induction can be rationally required rather than merely rationally permissible—which it is anyway in the absence of direct inference.

In sum, while an appeal to direct inference stands out as the most promising strategy for a probabilistic account of induction, neither the standard Bayesian framework nor that of logical probability can accommodate PNRC in a way that secures inductive inference. Thus the proponent of one of these standard probabilistic approaches needs to turn to some other implementation of direct inference in order to provide an account of induction. However, we shall see next that standard probabilistic approaches also struggle to successfully accommodate the main alternative to the Principle of the Narrowest Reference Class, namely David Lewis’ Principal Principle.

## The Principal Principle

Lewis (1980) uses single-case chances rather than generic frequencies to constrain prior probabilities:

- *Principal Principle.* $P_{\emptyset }(A| X E)=x$, where *X* says that the chance at time *t* of proposition *A* is *x* and *E* is any proposition that is compatible with *X* and admissible at time *t*.

Again, something needs to be said about when the additional evidence *E* is compatible with *X* and admissible, or instead defeats the application of the Principal Principle. Lewis specified that matters of fact up to time *t* are admissible, but remained non-committal about which other propositions are admissible.

The Principal Principle is immune to the problem for PNRC posed above, because reference classes have no bearing when the chances are single-case. However, the Principal Principle faces the following problem, due to Wallmann and Williamson (2020).

Suppose *E* is a proposition about matters of fact no later than the present, *A* says that it will rain tomorrow in Abergwyngregyn, and *X* says that the present chance of *A* is 0.7. The Principal Principle implies:

6.: $$P_{\emptyset }(A|X E) = 0.7$$.

Now consider an unrelated proposition *F*, which says that Fred’s fibrosarcoma will recur. Suppose the following assignment of probability is rationally permissible:

7.: $$P_{\emptyset }(F|X E) = 0.3$$.

In the standard Bayesian framework with the Principal Principle, such an assignment of degree of belief would be permissible as long as *E* does not determine that the chance of *F* is something other than 0.3. Such an assignment would also be permissible in the framework of logical probability, if *E* were to provide some relatively weak evidence against *F*. Suppose then that *E* provides at best weak evidence relating to *F*. In particular, suppose that *E* provides less compelling evidence than the present chance of *F*, in the sense that the present chance of *F* would trump *E* in determining strength of belief in *F*: $P(F|XYE) =y$, where *Y* says that the present chance of *F* is *y*.

Now consider the case in which *A* and *F* turn out to have the same truth value, i.e., $A\leftrightarrow F$. Conditional on $A\leftrightarrow F$, the propositions *A* and *F* must be given the same probability.^11^ What probability should that be? Since there is excellent evidence relating to *A*, namely the present chance of *A*, and at best weak evidence relating to *F*, it should at least be permissible that the probability of *A* is more strongly influenced by the present chance of *A* than by the weak evidence relating to *F*:

8.: $$P_{\emptyset }(A| X E (A\leftrightarrow F)) > 0.5$$.

The problem is that assignments 6-8 are inconsistent (Wallmann and Williamson 2020, §3.1). Arguably, then, neither the standard Bayesian framework nor that of logical probability can adequately accommodate the Principal Principle. Assignment 6 is just a simple application of the Principal Principle. Assignment 7 concerns an unrelated proposition. To violate assignment 8 would be to hold that an uninformed or weakly informed credence in an unrelated proposition should be as strong a determinant of your degree of belief in *A* as the present chance of *A*, where a conflict arises. This works against the intended goal of the Principal Principle, which is to ensure that credences are guided by chances. Moreover, to deny assignment 8 would be to maintain that although the present chance of *F* should trump *E* in determining strength of belief in *F*, $P(F|XYE) =y$, the present chance of *A* together with the fact that *A* and *F* have the same truth value should bizarrely have no special influence on strength of belief in *F*.^12^

It is worth observing that this problem for the Principal Principle also extends to the Principle of the Narrowest Reference Class. Suppose assignment 6 is generated by an application of PNRC with respect to a frequency in a very narrow reference class, instead of by an application of the Principal Principle: e.g., the frequency of rain in Abergwyngregyn after days on which the prevailing conditions are just like today’s. Suppose assignment 7, on the other hand, is induced by a frequency in a very wide reference class: e.g., the frequency of recurrence of fibrosarcoma in vertebrates. Then assignment 8 remains permissible. After all, the core idea of the Principle of the Narrowest Reference Class is that frequencies in narrower reference classes should prevail over those in wider reference classes when determining rational degrees of belief. However, assignments 6-8 are inconsistent. Hence, the standard Bayesian framework fails to accommodate simple rational belief assignments that are in line with the Principle of the Narrowest Reference Class.

So we see then that neither kind of direct inference principle sits easily in a standard Bayesian framework, because the direct inferences we might want (1-5 in the case of PNRC and 6-8 in the case of both the Principal Principle and PNRC) over-constrain the prior $P_{\emptyset }$, thanks to CBCP. Recasting direct inference in the framework of logical probability would not help, since logical probability presupposes CCCP, which is analogous to CBCP. One might therefore think that the prospects of any probabilistic approach to induction are dim.

However, this conclusion would be too hasty. There is a non-standard Bayesian approach that avoids the above problems by avoiding CBCP, as we shall see next.

## Objective Bayesian Inductive Inference

We noted in Sect. 2 that while strictly subjective Bayesianism leaves rational degree of belief largely unconstrained, empirically-based Bayesianism appeals to some direct inference principle to constrain rational degrees of belief given appropriate evidence. Objective Bayesianism holds that degrees of belief should be heavily constrained even in the absence of evidence. This is often achieved by means of the following principle. If $\Omega$ is a finite, indivisible set of mutually exclusive and exhaustive alternatives, then:

- *Maximum Entropy Principle.* $P_E$ is a probability function, from all those that satisfy constraints imposed by *E*, that maximises the entropy function,
  $$\begin{aligned} H(P) {\mathop {=}\limits ^{\mathrm{df}}} -\sum _{\omega \in \Omega } P(\omega ) \log P(\omega ). \end{aligned}$$

In the absence of any evidence, the Maximum Entropy Principle selects the equivocator function $P_=$, which gives each alternative the same probability, $P_=(\omega ) = 1/|\Omega |$ for all $\omega \in \Omega$. If there is substantive evidence, the Maximum Entropy Principle selects a probability function that is as equivocal as possible in the circumstances.

There are two versions of objective Bayesianism. The version most widely adopted is situated within the standard Bayesian framework and presumes CBCP. Some advocates of this version, particularly in the physical sciences, follow Jaynes (1957) in adopting the Maximum Entropy Principle, while others, particularly in the statistics community, follow Jeffreys (1939) in using other methods for determining ‘objective’ or ‘default’ priors. Either way, they can be considered advocates of what we shall call *standard* objective Bayesianism.

The alternative version of objective Bayesianism is that of Williamson (2010) and collaborators. This departs from the standard version in the following key ways.

Firstly, it rejects CBCP as a universal principle. While this move is a departure from standard Bayesianism, it does not amount to a rejection of probabilism. This is because the alternative version does take conditional beliefs to be probabilities:

- *CBP.* For any *E*, there is a probability function $P_{E}$ such that $B_E(A) = P_{E}(A)$ for all *A*.

By rejecting CBCP, this version of objective Bayesianism also eschews Conditionalisation as a norm that governs the updating of degrees of belief (Williamson 2010). In place of Conditionalisation, the Maximum Entropy Principle is used to constrain the choice of the belief function, on evidence *E*. This often gives results that agree with those produced by Conditionalisation, but not always. Use of the Maximum Entropy Principle has the advantage that it becomes possible to revise degrees of belief away from 0 and 1 in the light of unexpected evidence—something that it is not possible to do with Conditionalisation. In addition, the Maximum Entropy Principle can handle certain other cases that are problematic for Conditionalisation (Williamson 2010, §4.2).^13^

Second, while the standard Bayesian framework, which appeals to CBCP and Conditionalisation, requires that evidence be expressible in the domain of the probability function, i.e., in the algebra of $\Omega$, the alternative version of objective Bayesianism does not require this. This is advantageous because a framework in which the object language is not cluttered with all possible evidential propositions more accurately represents actual practice. In practice one cannot express all possible evidence, and even where one might be able to express one’s evidence it is often undesirable to do so. After all, we take propositions as evidence at least in part so they can be removed from the context of inquiry in order to focus on other propositions that are of immediate interest. Moreover, it is easier to represent and calculate probabilities defined over a smaller domain. Thus this approach to evidence leads to a more streamlined intellectual economy.

Third, this alternative version of objective Bayesianism incorporates direct inference. (Jaynes, in contrast, rejected the existence of non-epistemic probabilities.) The following direct inference principle is used to calibrate degrees of belief to single-case chances (Williamson 2021b):

- *Chance Calibration.* If, according to current evidence *E*, the current chance function $P^*$ lies in the set ${\mathbb {P}}^*$ of probability functions, then $P_E\in \langle {\mathbb {P}}^*\rangle$, the convex hull of ${\mathbb {P}}^*$.^14^

Here we take current evidence to be evidence about matters of fact up to the present. The qualification that *E* is current evidence is intended to ensure that *E* is admissible with respect to the present chance. Unlike the Principal Principle, this direct inference principle does not presuppose CBCP. Note that it requires a previous inference to a claim about the chance function. The agent needs to have established that $P^*\in {\mathbb {P}}^*$ from some previous body of evidence, and, having been established, the proposition $P^*\in {\mathbb {P}}^*$ is then included in the current body of evidence *E*.

Alternatively, degrees of belief can be calibrated to generic frequencies by means of some version of the Principle of the Narrowest Reference Class, such as the following:

- *Frequency Calibration.* If, according to *E*, the frequency $P^*_{\hat{\rho }}(\alpha )\in X$, and $\hat{\rho }$ is the unique narrowest reference class containing *c* with respect to which *E* determines non-trivial bounds on the frequency of $\alpha$, and *E* includes no more pertinent information, then $P_E(\alpha (c))\in \langle X\rangle$, the convex hull of *X*.

Again, this presumes some previous inference from data to the frequency proposition $P^*_{\hat{\rho }}(\alpha )\in X$.

The approach leaves open the question of whether the required inferences to chances or frequencies are performed using classical or Bayesian statistical inference. If the former, this version of objective Bayesianism can be thought of as marrying classical statistical inference (inference to generic frequencies or single-case chances) with Bayesian inference (inference from these non-epistemic probabilities to rational belief and action).

The fourth departure from Jaynes’ objective Bayesianism, as well as from the logical approach to probability, is that this version of objective Bayesianism does not require that rational degrees of belief be uniquely determined by the evidence. For example, the agent’s probabilities may be relative to the set $\Omega$ of indivisible alternatives as well as to the explicit evidence *E*. Here $\Omega$ can be thought of as determined by the agent’s language: if this language can be explicated by a finite propositional language ${\mathcal {L}}$ then $\Omega$ is the set of state descriptions of ${\mathcal {L}}$, and a similar account can be provided if ${\mathcal {L}}$ is a first-order predicate language (Williamson 2017). In addition, inferences from evidence to non-epistemic probabilities can depend on the agent’s utilities, as we note below. Finally, there may be multiple functions with maximum entropy, or indeed no maximum entropy function, in which case any function with sufficiently great entropy is rationally permitted, with what counts as ‘sufficient’ dependent on the agent’s interests. Thus, this version of objective Bayesianism relativizes probabilities to an agent’s language, utilities and interests, as well as evidence, and leaves some role for subjectivity.^15^ It should be thought of as a very highly constrained version of Bayesianism, but not uniquely constrained.

On the other hand, this version of objective Bayesianism is not over-constrained, as is standard Bayesianism with CBCP and the direct inference principles discussed in Sect. 3 and 4. This is because, without CBCP, direct inference constrains a different probability function $P_E$ for each body of evidence *E*. In contrast, standard Bayesianism with CBCP and either PNRC or the Principal Principle constrain a single prior probability function $P_{\emptyset }$. To get a sense of the extra degrees of freedom that this version of objective Bayesianism offers, observe that in this framework the five consequences of PNRC discussed in §3 would amount to:

$$1^\prime$$.
$$P_{XR}(A) = 0.17$$
$$2^\prime$$.
$$P_{YRS}(A) = 0.97$$
$$3^\prime$$.
$$P_{XYR}(A) = 0.17$$
$$4^\prime$$.
$$P_{XYRS}(A) = 0.97$$
$$5^\prime$$.
$$P_{XYR\bar{S}}(A) = 0.17$$.

Here, each of these consequences constrains a different probability function, so they cannot be mutually inconsistent. Similarly, the three conditions introduced in Sect. 4 translate as:

$$6^\prime$$.
$$P_{X E}(A) = 0.7$$.
$$7^\prime$$.
$$P_{X E}(F) = 0.3$$.
$$8^\prime$$.
$$P_{X E (A\leftrightarrow F)}(A) > 0.5$$.

While $6'$ and $7'$ constrain the same probability function, $P_{X E}$, they are consistent constraints on this function. Moreover, $8'$ constrains a different probability function, $P_{X E (A\leftrightarrow F)}$, and so cannot be incompatible with $6'$ and $7'$.

We see, then, that the inconsistencies of Sect. 3 and 4 simply do not arise in this alternative Bayesian framework.^16^

Having seen how this approach avoids the problems of Sect. 3 and 4, we next turn to the question of how it captures inductive inference.

If *A* is the atomic proposition $\alpha (c)$, the Maximum Entropy Principle mandates a middling degree of belief in *A*, $P_{\emptyset }(A) = 0.5$, in the absence of any evidence. Suppose the agent learns that a sample from reference class $\hat{\rho }$ yields proportion *x* for $\alpha$ (proposition *X*), and that individual *c* satisfies $\rho$ (proposition *R*). Suppose next that, from *XR*, the agent is prepared to use interval *I* as her best estimate of the chance $P^*(\alpha (c))$, i.e., she establishes that $P^*(\alpha (c))\in I$ (proposition *Y*) and is not prepared to commit to the chance lying in any narrower interval. Chance Calibration requires that her degree of belief in *A* should match this constraint on the chance, $P_{X Y R}(\alpha (c)) \in I$. The Maximum Entropy Principle then requires that she should choose a maximally equivocal value from within that interval—i.e., the value closest to 0.5:

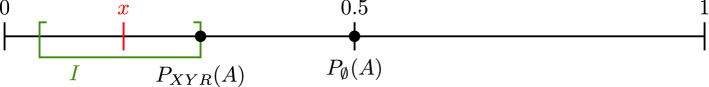



Williamson (2017, §7.3) breaks this inference down further into a series of small steps, by appealing to Frequency Calibration rather than Chance Calibration. These steps can be summarised as follows. Suppose $\hat{\rho }$ is the (unique narrowest) reference class of our sample *s* (e.g., 21-year-olds) and let $\hat{\sigma }$ be a reference class of similar samples (e.g., similar samples of a hundred 21-year-olds). Take $\bar{X}$ to be the function that maps a sample in $\hat{\sigma }$ to the mean of that sample; $\bar{X}(s)$ is thus the mean of our sample (e.g., 0.17, the proportion of members of the sample who get a cough). Then:

(i) Let $\tau$ be the threshold such that the agent would infer that $P^*_{\hat{\rho }}(\alpha )\in I$ should her credence in this proposition meet threshold $\tau$. $\tau$ can be determined from the agent’s utilities by means of Bayesian decision theory.^17^
(ii) Let $I_\tau$ be the function that maps a sample to the confidence interval determined by the sample mean and confidence level $\tau$.
(iii) One can infer that in approximately $100 \tau \%$ of samples, the corresponding confidence interval would capture the frequency $P^*_{\hat{\rho }}(\alpha )$, i.e., $P^*_{\hat{\sigma }}(P^*_{\hat{\rho }}(\alpha )\in I_\tau ) \approx \tau$.
(iv) Now consider our specific sample *s*, which is known to be in the reference class $\hat{\sigma }$ of similar samples. If there is no more pertinent evidence in *E* (including evidence gained from the sample itself), Frequency Calibration requires that $P_E(P^*_{\hat{\rho }}(\alpha )\in I_\tau (s)) \approx \tau$.
(v) By (i), the agent establishes that $P^*_{\hat{\rho }}(\alpha )\in I_\tau (s)$. This is added to *E* to give a new body of evidence $E^{\prime }$.
(vi) If $E^{\prime }$ also determines that individual *c* is a member of reference class $\hat{\rho }$, and $E^{\prime }$ contains no evidence more pertinent to *c*, then a second application of Frequency Calibration requires that $P_{E^{\prime }}(\alpha (c))\in I_\tau (s)$.
(vii) The Maximum Entropy Principle then further narrows down $P_{E^{\prime }}(\alpha (c))$ to the most equivocal value in $I_\tau (s)$.

This sequence of steps highlights the interplay between classical and Bayesian methods: step (i) appeals to Bayesian decision theory, (ii–iii) to classical statistics (frequentist confidence interval estimation methods), and (iv–vii) to the non-standard variant of objective Bayesianism.

In this alternative version of objective Bayesianism, then, direct inference ensures that rational degrees of belief are swayed by past experience and the Maximum Entropy Principle moderates the extent to which they are swayed. The exact extent to which past experience is moderated depends on the size of the confidence interval, which in turn depends on the confidence level, which is a function of the agent’s utilities. We will assess whether this account survives Schurz’ criticisms in the next section. But if successful, it provides a probabilistic account of the logic of induction that appeals to direct inference and is immune to the problems of Sect. 3 and 4.

## Schurz’ Criticisms

Schurz (2019, Sect. 4.5, 4.6) objects that this sort of account of induction requires a uniform prior probability distribution over the possible values of a frequency $P^*_{\hat{\rho }}(\alpha )$. He argues that this is a problem because equiprobability is language dependent—i.e., different languages lead to different distributions—and because uniform distributions prohibit induction. We will consider these three concerns—equiprobability, language dependence and induction being prohibited—in turn. I will argue that the non-standard objective Bayesian account of Sect. 5 is immune to these objections.

First note that this account does not require that evidence be included in the domain of the probability function, because it does not require CBCP. (This was the second difference between the two versions of objective Bayesianism noted in Sect. 5.) The upshot is that the agent’s language need not express frequency statements of the form $P^*_{\hat{\rho }}(\alpha )=x$, and objective Bayesianism does not require any prior probability distribution over the possible values of a frequency $P^*_{\hat{\rho }}(\alpha )$.

One might reply that, although this objective Bayesian approach does not actually require a uniform prior distribution over the frequencies, it is tantamount to one that requires such a distribution (see Maher 1996, §3). What this means is just that, if one were to try to emulate the non-standard objective Bayesian approach within the framework of standard Bayesianism, one would need such a prior distribution. This would in turn raise concerns about language dependence: the worry that a uniform distribution on one language may yield different inferences to a uniform distribution on another language.

However, to take this to be a problem is to assume that standard Bayesianism should have priority over this non-standard rival approach. For only then would it make sense to use the former approach to emulate the non-standard objective Bayesian approach. This begs the question. As I have argued above, we need to part from standard Bayesianism precisely in order to accommodate direct inference and induction.

Schurz provides the following example:

As an example, take a series of 100 coin tosses. It can be computed that with $p = 95$ percent the frequency of heads in 100 throws does not deviate by more than 8 percent from the true statistical probability of heads. Now assume we observe a number of 30 heads in 100 throws of the coin. According to Williamson’s argument we should now believe that the coin has a biased heads-probability of $30 \pm 8$ percent. That is only reasonable if our prior expectation concerning the coin’s true probability is uniform, which means that our prior expectation that the coin is approximately fair is very low. If we are confident that the coin is fair (i.e., our prior peaks about $p = \frac{1}{2}$), it seems more reasonable to believe that the given series was an unrepresentative accident. (Schurz 2019, 74)

It is certainly the case that, where there is evidence that the coin is fair, that evidence should influence an inference to frequency or chance. Even knowing that a coin is being tossed (as opposed to, say, merely knowing that an experiment is being conducted with at most two possible outcomes) provides some evidence against the probability of heads being close to 0 or 1. This evidence may be enough to resist the inference that the frequency or chance is in the 95% confidence interval. What exactly one should infer here about the frequency or chance is an open question. This scenario is clearly more complicated than the simple case in which there is no evidence that overrides the confidence interval estimate of the frequency or chance. But that does not undermine induction in the simple case, nor does it preclude induction in these more complex cases.

Thus Schurz’ conclusion is not warranted:

In conclusion, a justification of inductive posterior probabilities conditional on finite evidence solely by [direct inference], without assuming a particular prior distribution, is impossible. (Schurz 2019, 75)

Without CBCP, there is no need for any prior distribution of the probability of heads. Even if there is some prior distribution, it is the total evidence that guides degrees of belief, by means of direct inference and the Maximum Entropy Principle, not the prior distribution.

Let us turn to the question of language dependence. It is true that, in the absence of evidence, the Maximum Entropy Principle selects the equivocator function, which is a uniform distribution, and that objective Bayesianism ties probability to an agent’s language. But this is not a pernicious kind of language dependence because two different languages will agree on inferences that can be expressed in both languages (Williamson 2017, Theorem 5.9). Moreover, Bayesians should not be troubled by a link between probability and features of an agent such as her language. The whole idea of Bayesianism is to interpret probabilities as an agent’s rational degrees of belief and so relativity to features of an agent is inevitable. It is only the logical interpretation of probability that seeks to construe probability as an objective relation between propositions, determined solely by the propositions it relates. Admittedly, Jaynes gave great weight to probability distributions that are uniquely determined by the evidence and the problem formulation, in order to secure the objectivity of scientific inferences. But uniqueness is not essential to objective Bayesianism.

More serious is Schurz’ charge that uniform distributions prohibit induction. This is the worry, noted above, that the equivocator function fails to allow for learning from experience. It is a valid concern, but only under the presupposition of CBCP: if the prior probability function is the equivocator function, then conditionalising on a sample of a hundred ravens, all observed to be black, will not raise the probability of the next observed raven being black. This is not a valid concern for the version of objective Bayesianism advocated here, which rejects CBCP. From the sample of ravens one will infer that the frequency of ravens being black, or the chance of the next raven being black, is very close to 1, and direct inference ensures that one ought to believe that the next raven is black to some degree close to 1. Induction is by no means prohibited.

In sum, once we release ourselves from two dogmas of objective Bayesianism, namely CBCP and uniqueness, both of which are features of Jaynes’ account, Schurz’ criticisms lose their bite. The alternative version of objective Bayesianism can embrace both equiprobability and learning from experience, and is immune to Schurz’ criticisms.

## Conclusion

We saw in Sect. 2 that direct inference offers the most promising avenue for a probabilistic account of induction. However, neither the Principle of the Narrowest Reference Class nor the Principal Principle can realise this promise when situated within either of the two dominant probabilistic approaches, namely standard Bayesianism and logical probability. A non-standard variant of objective Bayesianism—that of Williamson (2010; 2017) offers a way out, though: it provides a probabilistic account of inductive inference that does not suffer from these problems that beset the standard approaches. A direct inference principle—Frequency Calibration and/or Chance Calibration—enables the account to accommodate learning from experience, while the Maximum Entropy Principle obviates the need for CBCP.

To be sure, there remain gaps in the account that need to be filled. Most notably, Chance Calibration leaves open the question of how to infer chances from evidence. Similarly, Frequency Calibration leaves open the analogous question, as well as that of how to determine whether *E* contains evidence that is more pertinent than the frequency and what to do when it does. We have seen how statistical techniques such as confidence interval methods can help to address these questions and how they slot into the logic of induction provided by this version of objective Bayesianism.

While a case can be made that this version of objective Bayesianism provides an inroad into the problem of inductive logic that is immune to Schurz’ criticisms, this is not to say that it offers the only satisfactory probabilistic account of induction. There are other non-standard probabilistic accounts, including the evidential probability account espoused by Kyburg and Teng (2001). Such accounts need not be rivals. In particular, evidential probability can be thought of as complementary to objective Bayesianism, because it can be construed as a way of implementing the Principle of the Narrowest Reference Class in objective Bayesianism—i.e, a way of filling the gaps in Frequency Calibration that we noted above (Wheeler and Williamson 2011).

The question remains as to whether the approach presented here offers any progress with regard to the problem of inductive justification. While this is not the place for a detailed consideration of this question, a few brief remarks may be helpful. As Schurz observes, its use of direct inference places this approach as a development of that of Williams (1947) and Stove (1986). However, this approach is distinctive in its appeal to Bayesianism: Williams and Stove advocated versions of logical probability (Peden 2021). In the approach presented here, inductive inference emerges as a consequence of the norms of objective Bayesianism, and it is justified to the extent that these norms are justified. Williamson (2010; 2017) argues along the following lines that these norms must be followed in order to avoid various kinds of loss. A standard Dutch book argument can be used to show that degrees of belief need to be probabilities in order to avoid sure loss (Williamson 2017, §9.2). Moreover, direct inference is required in order to avoid long-run loss, or expected loss (Williamson 2010, §3.3). Finally, degrees of belief need to conform to the Maximum Entropy Principle in order to avoid worst-case expected loss (Williamson 2017, §9.3). Thus, if a detractor from induction accepts that frequencies or chances govern the gains and losses that arise from one’s beliefs and decisions, and that one should avoid avoidable loss in one’s dealings with the world—including avoidable sure loss, long-run loss or expected loss, and worst-case expected loss—then this justification of objective Bayesianism may have some persuasive force.

This is perhaps a modest advance, as the detractor might resist an inference from a sample to a frequency or a chance in the absence of some justification that the sampling method is random. McGrew (2001) and Campbell and Franklin (2004) counter such scepticism, however, on the grounds that most large samples are representative of the population from which they are sampled, and (by direct inference) this fact warrants a default belief that the sample in question is representative, in the absence of evidence otherwise. If successful, this move shifts the burden of proof to the detractor.^18^

## Footnotes

1 Williams (1947, ch. 1) claims that the problem of inductive justification is not purely academic, suggesting that, Having, spiked the guns of reason, [the skeptic] has invited positive unreason to invade the citadel. All conscious and moral existence is a little clearing in the festering jungle of superstition, whose prowling terrors are fought off only by the courage and confidence of those who know what it is to know. Even within our circle now every doubt which unnerves the defenders of empirical reason is exploited by agents of the enemy, persons who are hostile to reason on principle: the logic haters, mystery lovers, and spell-binders. The obsequies of inductive logic are no sooner austerely announced by the skeptic than they are exultantly celebrated by enthusiasts reveling in the opportunity to advance some extra-scientific dispensation… (Williams 1947, 19) Williams overstates his case, however. Despite the long absence of any established justification of induction, inductive practice has grown hugely, ousting innumerable examples of superstition and quackery along the way.

2 The arguments of this paper do not hang on any particular definition of inductive inference, and no precise definition will be given. It will suffice to say that inductive inferences include inferences about a new case made partly on the basis of data or statistics about other cases. Examples will be provided below.

3 Karl Popper might be an exception (see, e.g., Rowbottom 2008). However, Popper was also an anti-inductivist—he argued instead for a falsificationist methodology. Consequently, we need not consider his view further here. Williamson (2000, ch. 10), Franklin (2001), Maher (2006), Mura (2008), Paris and Vencovská (2015) and Bird (2017, §5) are recent proponents of versions of logical probability.

4 More precisely, suppose ${\mathcal {L}}$ is a first-order predicate language with constant symbols $t_{1},t_{2},\ldots$ and atomic sentences $a_{1},a_{2},\ldots$, where the atomic sentences are ordered in such a way that sentences $a_{1},\ldots ,a_{r_n}$ involving only the first *n* constant symbols appear before those involving $t_{n+1}$, for each *n*. The *n*-states $\omega _{n}\in \Omega _{n}$ are the sentences of the form ${\scriptstyle \pm }a_{1}\wedge {\scriptstyle \pm }a_{2}\wedge \cdots \wedge {\scriptstyle \pm }a_{r_n}$ where ${\scriptstyle \pm }a_{i}$ is either $a_{i}$ or $\lnot a_{i}$ for each $i=1,\ldots ,r_n$. These are the state descriptions involving only the first *n* constants. For each *n*, the equivocator function gives each *n*-state the same probability.

5 Carnap acknowledged the limitations of his approach and later became more sympathetic to a Bayesian approach (see Carnap 1962; 1968).

6 More complicated versions of the Principle of the Narrowest Reference Class say something about what to do in the presence of statistics from multiple narrowest reference classes (see, e.g., Thorn 2019).

7 The following argument is cast in terms of the Bayesian framework because that is the usual setting for direct inference. However, similar points can be made about the logical framework, on account of its appeal to the analogous principle CCCP. Carnap (1950, §94) and Paris and Vencovská (2015, ch. 15), for example, argue that their versions of the logical theory validate direct inference.

8 This kind of inconsistency was identified by Wallmann and Hawthorne (2020, §5.2), though neither author takes it to be of concern (personal communication). I will argue that the inconsistency leads to a problem that is decisive in this context.

9 Here $P^*$ is the frequency function relative to the trivial reference class, which contains all individuals as members. See for example Gillies (2000, ch. 5) for an account that takes frequencies to be conditional probabilities.

10 In order to make $\sigma$ more concrete, one might try to construct a suitable reference class. For instance, consider the reference class $\sigma$ consisting of those individuals not called ‘Cheesewright’ who get a cough in the next year. We know that this reference class makes a positive difference: $P^*_{\widehat{\rho \sigma }}(\alpha ) = 1$ and $P^*_{\widehat{\rho \bar{\sigma }}}(\alpha ) \approx 0$, and that Cheesewright is not a member of $\sigma$, i.e., $\bar{S}$. So we might consider $P_{\emptyset }(A|XYR\bar{S})$ instead of $P_{\emptyset }(A|XR)$. Now, $P_{\emptyset }(A|XYR\bar{S}) \approx 0$, not 0.17. Thus $Y\bar{S}$ apparently defeats our proposed use of PNRC. However, as Thorn (2012) discusses, there are independent reasons to avoid gerrymandered reference classes when applying the Principle of the Narrowest Reference Class.

11 This is a consequence of the axioms of probability: for any probability function *P*, $P(A|A\leftrightarrow F) = P(A\wedge F|A\leftrightarrow F) + P(A\wedge \lnot F|A\leftrightarrow F) = P(A\wedge F|A\leftrightarrow F) + 0 =P(A\wedge F|A\leftrightarrow F) + P(\lnot A\wedge F|A\leftrightarrow F) = P(F|A\leftrightarrow F)$.

12 One might suggest moving from Lewis’ Principal Principle to some other principle that calibrates degrees of belief to chances. The main alternative is a version of the Principal Principle that appeals to conditional chance (Hall 1994; 2004):

- *Conditional Principal Principle.* $P_{\emptyset } (A|XE)=x$, where *X* says that the chance at *t* of *A*, *conditional on* *E*, is *x*.

However, Wallmann and Williamson (2020, §3.2) show that the Conditional Principal Principle does not avoid the problem for the Principal Principle outlined above. See Wallmann and Williamson (2020) for more detail and for responses to further suggestions for avoiding the above problem.

13 Kyburg (1977) also rejects Conditionalisation, in response to an objection of Levi (1977) that Kyburg’s implementation of the Principle of the Narrowest Reference Class conflicts with Conditionalisation—see also Seidenfeld (2007, §3) on this point. While Kyburg’s theory is usually classed as non-Bayesian, the approach presented here is more naturally classed as a Bayesian theory, albeit non-standard. This is because it takes probabilities to be rational degrees of belief and because updating by maximising entropy often produces results that agree with Conditionalisation. This approach can be viewed as a development of Bayesianism that resolves certain difficulties with the standard approach. Kyburg’s theory of evidential probability, on the other hand, is closer to logical probability, and does not appeal to the Maximum Entropy Principle.

14 Recall that a probability function *P* is in the convex hull of a set of probability functions iff it is a mixture of probability functions in that set, i.e., iff there are functions *Q* and *R* in the set and some real number $\lambda$ in the unit interval such that $P(\omega ) = \lambda Q(\omega ) + (1-\lambda ) R(\omega )$ for each $\omega \in \Omega$.

15 Indeed, this version of objective Bayesianism arguably handles subjectivity in a better way than does the standard Bayesian framework. Suppose at least two degrees of belief in *A* are rationally permissible, *x* and *y*, and then some evidence irrelevant to *A* is obtained. In the standard Bayesian framework, one needs to select one of these degrees of belief—*x* say—and conditionalise on the new evidence, after which degree of belief *x* must be retained. This flies in the face of common sense: if *y* was rationally permissible before learning the irrelevant evidence, it should still be rationally permissible. Common sense is salvaged by updating via the Maximum Entropy Principle rather than Conditionalisation. If both *x* and *y* satisfy constraints imposed with the evidence before the arrival of the new information, and have sufficiently great entropy, then they will also be compatible with the evidence and have sufficiently great entropy after adding the irrelevant information. Thus both remain rationally permissible, as required.

16 Does that mean that $1'-8'$ should all hold in this setting? Recall that 5, although intuitively plausible, is questionable, for the reasons discussed in Sect. 3: as Eq. ii shows under certain assumptions, the frequency of getting a cough in the reference class of those without tuberculosis must be less than 0.17. This argument against 5 also brings $5'$ into question: given that the frequency is not 0.17, is it reasonable to believe that Cheesewright gets a cough to degree 0.17? While we do not need to settle this question here, we should note that denying $5'$ would open the door to the mischief-maker argument of Sect. 3. Thus some further account would need to be given of how to avoid the mischief maker. The key point here is that there is no inconsistency in this alternative framework that compels one to deny any of $1' - 5'$. The Bayesian can endorse all these applications of the Principle of the Narrowest Reference Class, by moving from standard Bayesianism to the non-standard objective Bayesian framework.

17 More precisely, $\tau = \frac{S_{2}-E_{2}}{S_{1}+S_{2} - E_{1} - E_{2}}$, where $S_{1}$ is the utility of establishing the interval estimate $P^*_{\hat{\rho }}(\alpha )\in I$ if the estimate is correct, $S_{2}$ is the utility of not establishing it if it is incorrect, $E_{1}$ is the utility of not establishing it if correct and $E_{2}$ is the utility of establishing it if incorrect. See Williamson (2021a) for further discussion of this point.

18 Maher (1996) for one, although a critic of the Williams-Stove approach, argues that it is unreasonable for a detractor to insist on evidence of randomness.
